# The TOUCH program and natalizumab: Fundamental flaw in patient protection

**DOI:** 10.12688/f1000research.7513.3

**Published:** 2016-05-10

**Authors:** Jagannadha Avasarala

**Affiliations:** 1 Department of Medicine/Division of Neurology, Greenville Memorial Hospital & Neuroscience Associates, Greenville, SC, 29615, USA

**Keywords:** natalizumab, multiple sclerosis, progressive multifocal leukoencephalopathy, TOUCH, John Cunningham Virus antibody, JCV Ab

## Abstract

Many drugs have been approved by the Food and Drug Administration (FDA) since 1993 for treatment of relapsing forms of multiple sclerosis (MS). One such drug is natalizumab (Tysabri, Biogen Idec and Elan pharmaceuticals) which has enjoyed great success in the management of MS since its re-introduction in 2006. One of the complications of using natalizumab is the risk of development of progressive multifocal leukoencephalopathy (PML). To mitigate the risk of PML development, Biogen Idec initiated the TOUCH program – this strategy helps monitor the disease. Clinical vigilance remains key in the early diagnosis of PML but serological testing for the John Cunningham Virus Antibody (JCV) helps with risk stratification of PML. However, some physicians do not test for the JCV Ab and since they are not required to send such data to the company or inform the patient, one red flag for suspicion of PML is lost particularly if the patient is asymptomatic.  This undercuts the premise of the TOUCH program. In an ideal world, reporting JCV Ab status should be made mandatory since that ensures a basic tenet of the program is met – to identify patients at increased risk of developing PML and make appropriate recommendations based on that finding. Lack of requirement of reporting of this vital finding opens the door for uncertainty in assessment of risk PML development and everyone remains in the dark till it may be too late. This is unacceptable when the company created the TOUCH program specifically with intent to track PML risk in patients on natalizumab. It makes no scientific sense to let the drug be used without setting stringent criteria given the possibility of PML development.

Natalizumab is the first monoclonal antibody approved for the treatment of relapsing forms of multiple sclerosis (MS) and is used in more than 50 countries. Natalizumab is a recombinant humanized monoclonal IgG4 antibody that binds to alpha4 subunit of the alpha 4 beta 1 integrin molecule expressed on leukocytes (except neutrophils) and inhibits the alpha4-mediated adhesion of leukocytes to extracellular matrix, endothelial lining, vascular cell adhesion molecule (VCAM 1) and fibronectin. After its initial approval in 2004 by the FDA, it was voluntarily withdrawn in early 2005 after two patients with MS in the SENTINEL trial and 1 patient with Crohn’s disease were diagnosed with progressive multifocal leukoencephalopathy (PML) ^1– 3^.

The drug was reapproved in 2006 and recommendations were made in the US to limit its use in highly active relapsing-remitting MS (with more than two relapses per year) and in those patients who do not respond or tolerate first-line treatment such as interferon beta-1a, interferon beta-1b, or glatiramer acetate. As well, a restricted risk minimization plan was initiated to better assess an individual’s risk of PML: Tysabri Outreach: Unified Commitment to Health [TOUCH]. This created a system where only prescribers and patients enrolled in the TOUCH program could prescribe and receive the drug. Only pharmacies and infusion sites authorized by the TOUCH prescribing program can dispense and infuse natalizumab. The primary goals of the program are to inform prescribers, infusion center healthcare providers and particularly patients, about the risk of development of PML associated with natalizumab use including the positive association of increased risk of PML with a) treatment duration, b) prior immunosuppressant use and c) positive JCV Ab status. The TOUCH program also includes information on, and warnings against, concurrent use of natalizumab with antineoplastic, immunosuppressant, or immunomodulating agents and in patients who are immunocompromised. In 2012, the FDA approved the STRATIFY JCV Ab ELISA test, a qualitative test to detect the presence or absence of JCV antibodies. Since this test adds to PML stratification and risk evaluation in natalizumab users, Biogen’s TOUCH program questionnaire requires physicians to indicate the JCV Ab status of each patient after every 6 months of use of the drug; however, testing for JCV Ab is not mandatory and some physicians do not order the test, thus endangering patient safety. This runs counter to the premise of the Risk Evaluation and Mitigation Strategy (REMS) under which the TOUCH program was commissioned by the FDA and it is time for Biogen to plug that loophole since it is aware that this is occurring.

Additionally, the FDA has not approved the validity or applicability of the JCV Ab index (anti-JCV Ab levels in serum/plasma) which may differentiate PML risk in JCV-Ab positive MS patients with no prior immunosuppressant use ^4^. Despite its lack of FDA approval status, the JCV Ab index is widely used by MS clinicians in the risk evaluation of PML development. Clinicians tend to worry once the index begins to rise although doubling the index value, for instance, does not automatically confer twice the risk of PML development. Since the index is not FDA-approved, the TOUCH program cannot mandate its routine use but every patient who has some basic understanding of the PML saga in MS wants to know his/her JCV Ab index. Laboratories run the test, clinicians use it for better or worse and yet the TOUCH program cannot adopt it. It is not an inherent flaw of the TOUCH program itself but sooner rather than later, the FDA should establish whether the JCV Ab index is valid and whether it can be part of a modified TOUCH program or not.

Another confusing test that some clinicians continue to use without rhyme or reason and on a monthly basis is the measurement of JCV DNA viremia ^5^. This too, akin to the JCV Ab index, is not part of the TOUCH program risk assessment strategy for PML. Although viremia by itself is not a predictor of PML risk, that it can occur in JCA Ab negative patients ‘raises other issues’ according to authors who advocate ‘periodic monitoring’ over the course of the treatment with natalizumab without offering specific time-specific testing protocols ^5^. Again, the TOUCH program administrators cannot be responsible if testing for JCV viremia does not have scientific relevance and if uninformed clinicians continue to pursue JCV DNA studies religiously, falsely assuming that they are tracking PML – they are not. The test is superfluous and literally a waste of patient’s blood and money.

Most clinicians track PML using JCV Ab status every 6 months as required but as a neurologist and a fellowship-trained multiple sclerosis physician, I have seen patients without JCV Ab testing or reporting who continue to be in the TOUCH program. It is also true that JCV Ab status, if positive, does not imply PML development, but it begs the question as to why the TOUCH program does not insist that JCV Ab status be reported every 6 months. A simple solution would be to make the JCV Ab status available to the company which then absolves Biogen from any culpability or negligence; if the patient and their physician opt to continue the drug despite JCV Ab status being positive, that is a choice between the two parties. Obviously, JCV Ab positive status is one of many factors that can increase the risk of PML development – use of the drug beyond two years and prior immunosuppressant use also increase the risk of PML. Clinicians understand and agree that early diagnosis of PML hinges on clinical vigilance.

Since Biogen Idec and the FDA are interested in halting PML in its tracks, and there have been, as of March 2016, a total of 635 confirmed cases of PML in MS while on natalizumab ^6^, it must be obvious for all those concerned with patient safety that it is necessary to screen for JCV Ab status in patients at 6 monthly intervals. Strangely enough, confirmed PML cases from natalizumab use are not available in a database for researchers to probe into individual (personal details can be encrypted) cases for analysis. The primary goal of the TOUCH program is to address risk stratification of PML and therefore, allowing clinicians to continue to prescribe natalizumab without knowledge of the JCV Ab status is a huge risk. It would be an easy recommendation to make JCV Ab testing mandatory; making JCV Ab status reporting the *sine qua non* for prescribing this drug adds one more layer of protection to patients.

It is unknown if any of the 635 reported cases of PML fall into the category that I have described – even if only one patient did, this would call into question whether it was preventable and what the role of the TOUCH program should have been in preventing it. One wonders what proportion of patients do not have their JCV Ab status reported across the globe while in the TOUCH program. Since hundreds of PML cases are already known, and more will likely continue to be reported, it is conceivable that questions will be raised as to whether more could have been done to prevent such cases. I hope there are no instances of PML owing to omission of JCV Ab status evaluation but I also think it is time for FDA to act *now* to prevent future lapses and avoid legal nightmares. My suggestion would be to make reporting of JCV Ab status mandatory for all patients on natalizumab in the TOUCH program - from a pharmacovigilance perspective, this makes perfect sense.
