# Supplementary material for: The TOUCH program and natalizumab: Fundamental flaw in patient protection
Source: F1000Res. 2016 May 10;4:1450. Originally published 2015 Dec 15. [Version 3] doi: 10.12688/f1000research.7513.3 (PMC4837976; doi:10.12688/f1000research.7513.3)
Supplement: Peer review history

# Peer review: Referee response for version 2

**DOI:** 10.5256/f1000research.8711.r13286

**Published:** 2016-04-19

**Authors:** Michael Levy

**Affiliations:** 1 Department of Neurology, Johns Hopkins University, Baltimore, MD, USA

This is a thoughtful article from the patient care point of view that is focused on the best interest of patients. With the increasing incidence of PML with natalizumab, I agree with the author's opinion that routine JCV antibody testing should be required in the TOUCH program. I also agree with the the author that the JCV antibody testing will be a part of the whole clinical picture in deciding whether or not to continue natalizumab treatment for any individual patient.

I have read this submission. I believe that I have an appropriate level of expertise to confirm that it is of an acceptable scientific standard.

# Peer review: Referee response for version 2

**DOI:** 10.5256/f1000research.8711.r12574

**Published:** 2016-03-30

**Authors:** Nicholas Schwab

**Affiliations:** 1 Department of Neurology, University Hospital Münster, Münster, Germany

I have read the revised version of Dr. Avasarala and I think the reasoning behind the article is sound. For absolute patient safety it is necessary to check a patient's anti-JCV antibody status and most physicians follow that rule.

However, I stand by my opinion that there are singular cases, where a patient might not want to know their status, because a treatment with natalizumab is the only choice they have and they do not want to be worried, if they have to be treated with natalizumab anyway. I acknowledge that for some the personal choice is not as important as the overall patient safety, but for me it is.

Additionally, the introduction of the JCV serology has not lead to dramatically reduced PML incidence rates and almost all current PML cases have had their status checked before - and opted to continue their treatment. I do not see this changing with mandatory status assessments and would still state that the test needs to be available for everyone who wants to use it.

As the prerequisite for this availability is the perfectly educated physician, I would argue that the initial prescription of natalizumab might be restricted to specialists, who have that education. This might already be enough to erase safety concerns and make an addition to the labelling unnecessary.

I have read this submission. I believe that I have an appropriate level of expertise to confirm that it is of an acceptable scientific standard.

# Response

**Published:** 2016-04-05

**Authors:** Jagannadha Avasarala

**Affiliations:** Greenville Hospital System University Medical Center, USA

I agree with Dr Schwab regarding his revised comments. The point of my article is simple - Natalizumab continues to be used by specialists and non-specialists alike and some of them, the physicians, do NOT obtain JCV Ab titers as they are supposed to. After STRATIFY became available and FDA approved its use, in 2012, all Biogen had to do was to introduce it as one more safety measure in the TOUCH program and streamline the process. Instead, the company allows, to this day, physicians to continue to prescribe the drug sans testing or reporting of the JCV Ab status and that undercuts the principle of the TOUCH program, designed to monitor or track PML (risk assessment). 

Following is what the FDA said in 2012, on their website

**FDA permits marketing of first test for risk of rare brain infection in some people treated with Tysabri**

Today, the U.S. Food and Drug Administration allowed marketing of the first test to help ***determine the risk ***for a rare brain infection called progressive multifocal leukoencephalopathy (PML) in people using the drug Tysabri (natalizumab) to treat multiple sclerosis (MS) or Crohn’s disease (CD).

The Stratify JCV Antibody ELISA test, ***when used with other clinical data*** from the patient, can ***help health care providers determine the risk for developing PML ***in MS and CD patients.

Dr Avasarala

# Response

**Published:** 2016-04-08

**Authors:** Jagannadha Avasarala

**Affiliations:** Greenville Hospital System University Medical Center, USA

Just to keep readers updated, the PML count is now 635, as of March, 2016. This is the # of ****patients with PML who are on Tysabri for MS.

Dr Avasarala

# Peer review: Referee response for version 1

**DOI:** 10.5256/f1000research.8092.r12282

**Published:** 2016-02-09

**Authors:** Nicholas Schwab

**Affiliations:** 1 Department of Neurology, University Hospital Münster, Münster, Germany

The first point I would like to make (as it is mentioned in the abstract) is that TOUCH has not been introduced by Biogen as a method to mitigate PML risk, but as a method to inform patients and physicians of the PML risk and to monitor the patients for early signs of PML allowing for a better diagnosis and treatment. While some might argue that PML prevention ***should*** be the ultimate goal of TOUCH, it has not been designed to do that and one can hardly blame the program for not reaching a goal it has not been set up to achieve.

The FDA approval of the JCV index should indeed be pursued, as the author is right in assuming that patients and physicians already use the JCV index in risk stratification decisions and the sooner the FDA rules on the biomarker, the better, so it can be applied during TOUCH in a coordinated and sensible way.

The data concerning JCV viremia and PML risk does not support it as a risk biomarker and I would either downright state that or remove the paragraph.

I agree with the author that the consequent monitoring and application of the JCV serology would be a step towards reducing PML incidence, as JCV serology is still the most sensitive biomarker with regard to PML development. However, I would personally say that it is up to patient and physician to either use the serology or choose not to. While the goal of maximum safety is a commendable one, I would argue that personal choice on whether a patient wants to know their JCV status is even more important. It would be a different situation, if the JCV serology had a high specificity, then the use of natalizumab should be restricted to anti-JCV negative patients. With a low specificity of ca. 45% it can be reasoned that a patient does not want to know their status, if they urgently need to use natalizumab anyway and prefer not be worried about their PML risk.

While it would be a great data resource to know and monitor the JCV serostatus (and potentially index) of all TOUCH patients, to force a biomarker with low specificity on patients, who might choose not to use it, would have far-reaching consequences. The knowledge of their JCV serostatus has not prevented the occurrence of the 300+ PML, where it was available before, so I do not think that the mandatory use would help in this regard. The biomarker should, however, be available to all patients, who want to use it, so no PML cases develop, where the patient was unaware of their possibility to test for anti-JCV antibodies. To my knowledge, this is already the case. The TOUCH program should be updated in the future to include possible alternative biomarkers as well and serve as a monitoring platform.

Having said that, I fully support the author’s with for a usable database, where physicians and researcher can access the data of the PML patients for research purposes to get a better handle on this devastating disease.

I have read this submission. I believe that I have an appropriate level of expertise to confirm that it is of an acceptable scientific standard, however I have significant reservations, as outlined above.

# Response

**Published:** 2016-02-09

**Authors:** Jagannadha Avasarala

**Affiliations:** Greenville Hospital System University Medical Center, USA

I thank Dr Schwab for his insight and comments on my article. Here are my responses, itemized.

1. To quote the TOUCH program official website statement *verbatim*, under the sub-heading of 'a commitment to patient safety', the following is noted - 

- Because of the risk of PML, TYSABRI® (natalizumab) is available only through a restricted program under a *Risk Evaluation and Mitigation Strategy (REMS) called the TOUCH® Prescribing Program*.

2. One has to also note that REMS, an FDA mandated program, noted the following for TYSABRI use - To inform prescribers, infusion center healthcare providers, and patients about the risk of progressive multifocal leukoencephalopathy (PML) associated with TYSABRI including the a) increased risk of PML with longer treatment duration, b) prior immunosuppressant use and c) *the presence of anti-JCV antibodies.*

The above mentioned strategies came to a head with the development of STRATIFY, a test developed to stratify PML risk, and approved by the FDA in 2012 to monitor PML risk. As well, Biogen has a questionnaire that all healthcare providers enrolled in the TOUCH program have to complete every 6 months *that includes a section regarding the JCV Ab status.* My question is simple - what is the point of the TOUCH program, STRATIFY test, FDA approval of risk mitigation strategies, inclusion of the JCV Ab status in the questionnaire, etc., if physicians are allowed to *discard the very test that is supposed to protect a patient by stratification of risk* as defined by the guidelines ? Clinical surveillance, frequent MRI evaluations, history of use of other immuno-suppressant drugs in the past, as well as duration are all factors that drive PML risk higher but what of the company that put all the pieces of risk evaluation in the first place ? One cannot walk away from the basic tenet in this discussion with semantics - patient protection from PML in TYSABRI users. From 2012 and beyond, after the STRATIFY was developed, there is no excuse for Biogen to let physicians prescribe TYSABRI without checking for JCV Ab status and certainly one way of reassuring the medical community would be to a) make testing mandatory for TYSABRI continuation and b) make the PML database open to researchers to investigate if cases were indeed missed as a result of this simple error. 

Jagannadha Avasarala

# Peer review: Referee response for version 1

**DOI:** 10.5256/f1000research.8092.r11563

**Published:** 2016-02-03

**Authors:** David B Clifford

**Affiliations:** 1 Department of Neurology, Washington University in St. Louis, St Louis, MO, USA

I think that advocating to legislate science that is admittedly not yet accepted as useful is not such a good idea.  Changing the goal to driving data collection to decide risk stratification that avoids PML might be a good idea.  TOUCH was not created to provide risk stratification, but to help assure early diagnosis.  If there is any enhancement, it might be better to advocate for frequent MRI which do appear to improve outcomes for PML.  To date, there is no prospective evidence that antibody monitoring prevents PML, and indeed if anything the evidence is that it does not, since cases continue while it is available.  I would suggest re-working the recommendation to a program that helps prove if antibody data actually can help physicians prevent PML. 

I recommended removing the distracting paragraph about JC DNA.

I would recommend including frequency of imaging as part of TOUCH since it appears to help make earlier diagnosis and improve outcomes.

I think questioning whether TOUCH is effective at present is realistic.

I have read this submission. I believe that I have an appropriate level of expertise to confirm that it is of an acceptable scientific standard, however I have significant reservations, as outlined above.

# Response

**Published:** 2016-02-03

**Authors:** Jagannadha Avasarala

**Affiliations:** Greenville Hospital System University Medical Center, USA

I thank Dr Clifford for his erudite observations. 

1, I agree that the TOUCH program helps in early diagnosis - **in fact, the following are explicitly stated on the touchprogram.com, thus:**

 

- Inform prescribers, infusion center healthcare providers, and patients about the risk of progressive multifocal leukoencephalopathy (PML) associated with TYSABRI including the increased risk of PML with treatment duration and prior immunosuppressant use.
- Warn against concurrent use with antineoplastic, immunosuppressant, or immunomodulating agents and in patients who are immunocompromised.
- **Promote early diagnosis of PML** and timely discontinuation of TYSABRI in the event of suspected
- Furthermore, in the important safety information section, it is clearly noted that risk factors for the development of PML include duration of therapy, prior use of immunosuppressants, ***and presence of anti-JCV antibodies.***

As clinicians, we all know that 'early diagnosis' of PML includes the STRATIFY testing protocol approved by the FDA in 2012 and that test is designed specifically to assess PML risk. We also understand and know that JCV Ab negative status also carries risk of PML development but what we cannot ignore what the safety information (noted above) and STRATIFY testing are designed to do. Therefore, uncoupling the TOUCH program from testing/reporting JCV Ab status is not only dangerous and fallacious, it runs counter to the argument that there is any 'risk stratification' being done if this simple testing is forgotten or discarded. 

No one can claim that JCV Ab monitoring prevents PML and nor do I state that; but assessing PML risk with STRATIFY is a fundamental principle of the test or we could discard the test altogether ! That frequent MRI testing, clinical surviellance (most critical) and patient self-reporting of new symptoms or worsening of existing symptoms is paramount to the diagnosis of PML is a well established fact, based on scientific evidence.

**If JCV Ab testing is of such low importance, why does the TOUCH program questionnaire include this as part of their questionnaire ?** One cannot have it both ways. Either the testing is critical or we do not test it at all and shun the JCV Ab testing as well as the JCV index. Why have a test approved by the FDA (Stratify), create the TOUCH program to monitor and track PML, include JCV Ab status in the questionnaire that is generated by the company and yet reject the very idea of monitoring for PML by throwing away the JCV Ab testing ? 

The statement that "TOUCH program was created to help assure early diagnosis" utterly does not hold water if JCV Ab testing is not done. As pointed out in my paper, the questionnaire itself includes it ! What is the inclusion for ? It is not exactly for statistical purposes, is it ?

Dr Avasarala, MD, PhD

# Response

**Published:** 2016-02-03

**Authors:** Jagannadha Avasarala

**Affiliations:** Greenville Hospital System University Medical Center, USA

I would also submit the following, additional comments.

The TOUCH program, when it was first introduced, did not have the benefit of STRATIFY, approved in 2012. But once JCV Ab testing was FDA approved in 2012, and JCV Ab status testing was part of the TOUCH questionnaire to continue Tysabri use, it became an essential tool to monitor PML risk (in fact, the word Stratify is itself a connotation to categorize risk of PML) so how it is part of a strategy to assess PML risk and yet can be ignored at the same time does not add up. If patients are to be protected or their risk explained to them, every single tool available needs to be put to use. Simple as that.
